# Understanding human aging and the fundamental cell signaling link in age-related diseases: the middle-aging hypovascularity hypoxia hypothesis

**DOI:** 10.3389/fragi.2023.1196648

**Published:** 2023-06-13

**Authors:** Teow J. Phua

**Affiliations:** Molecular Medicine, NSW Health Pathology, John Hunter Hospital, Newcastle, NSW, Australia

**Keywords:** healthspan, aging, hypoxia, oxidative stress, inflammation, nitric oxide, hypoxia-inducible factor, oxygen sensing

## Abstract

Aging-related hypoxia, oxidative stress, and inflammation pathophysiology are closely associated with human age-related carcinogenesis and chronic diseases. However, the connection between hypoxia and hormonal cell signaling pathways is unclear, but such human age-related comorbid diseases do coincide with the middle-aging period of declining sex hormonal signaling. This scoping review evaluates the relevant interdisciplinary evidence to assess the systems biology of function, regulation, and homeostasis in order to discern and decipher the etiology of the connection between hypoxia and hormonal signaling in human age-related comorbid diseases. The hypothesis charts the accumulating evidence to support the development of a hypoxic milieu and oxidative stress-inflammation pathophysiology in middle-aged individuals, as well as the induction of amyloidosis, autophagy, and epithelial-to-mesenchymal transition in aging-related degeneration. Taken together, this new approach and strategy can provide the clarity of concepts and patterns to determine the causes of declining vascularity hemodynamics (blood flow) and physiological oxygenation perfusion (oxygen bioavailability) in relation to oxygen homeostasis and vascularity that cause hypoxia (hypovascularity hypoxia). The middle-aging hypovascularity hypoxia hypothesis could provide the mechanistic interface connecting the endocrine, nitric oxide, and oxygen homeostasis signaling that is closely linked to the progressive conditions of degenerative hypertrophy, atrophy, fibrosis, and neoplasm. An in-depth understanding of these intrinsic biological processes of the developing middle-aged hypoxia could provide potential new strategies for time-dependent therapies in maintaining healthspan for healthy lifestyle aging, medical cost savings, and health system sustainability.

## Introduction

Aging-related hypoxia, oxidative stress, and inflammation pathophysiology are closely associated with human age-related carcinogenesis and chronic diseases (Muz et al., 2015; Göbel et al., 2021; Mas-Bargues et al., 2021; Bouhamida et al., 2022; Gambini and Stromsnes, 2022; Leyane et al., 2022; Wei et al., 2022). However, the connection between hypoxia and hormonal cell signaling pathways is unclear (Yang et al., 2018; Tran et al., 2020; Jehanno et al., 2022), but such human age-related comorbid diseases do coincide with the middle-aging period of declining sex hormonal signaling (Horstman et al., 2012; Diamanti-Kandarakis et al., 2017; Khadilkar, 2019). The middle-aging period is between the fifth (40s) and before the seventh (60s) decade of life (Phua, 2021). Aged-related comorbid diseases (Franceschi et al., 2018; Laconi et al., 2020) are a global burden (Kocarnik et al., 2022) with consequences for future health system sustainability (Soerjomataram and Bray, 2021). In one study of cancer-related deaths in the United States, 90% of cancers were diagnosed in the aged group of those over 50 years (Siegel et al., 2022).

Current knowledge shows that age-related diseases in humans are complex and heterogeneous in nature. This scoping review (Munn et al., 2018; Sargeant and O’Connor, 2019) aims to evaluate the relevant interdisciplinary (Greenwald and Dunn, 2009; Okamura, 2019; Cherbuin et al., 2021) evidence that has been accrued to assess the systems biology (Wang et al., 2018; Batie et al., 2022; Zhang et al., 2022) of function, regulation, and homeostasis in order to discern and decipher the etiology of the connection between hypoxia and hormonal signaling in human age-related comorbid diseases. It requires a descriptive approach to provide a series of stepwise, evidence-based functional interactions between the interdisciplinary modules in constructing the hypothesis of hypovascularity hypoxia.

Altogether, this new approach and strategy (Brennan and Davey-Smith, 2022; Diokno, 2022; Zhang et al., 2022) can provide clarity of concepts and patterns to determine the causes of declining vascularity hemodynamics (blood flow) and physiological oxygenation perfusion (physoxia) related to oxygen homeostasis and vascularity that cause hypoxia (hypovascularity hypoxia).

The prostate aging degeneration hypothesis postulates that this triad of testosterone, vascular, and inflamm-aging results in the conjoining of nitric oxide downregulation and vascular/endothelial dysfunction and inflammation, leading to age-related dysfunctions of amyloidosis and autophagy within an evolutionary tumorigenesis microenvironment (Phua, 2021). The earlier author’s short text findings, published in the journal Medicines by MDPI, form the basis for the discussion on prostate aging degeneration, and the crosstalk between testosterone, vascular, inflamm-aging, p53, cellular senescence, amyloidosis and autophagy below.

This integrative scoping review (Munn et al., 2018; Sargeant and O’Connor, 2020) would evaluate the importance of vascular function from the prostate aging degeneration hypothesis (Phua, 2021) in unlocking the biological secrets of aging (Borrás, 2021) and for the non-mutagenic promoters in carcinogenesis (Brennan and Davey-Smith, 2022). Both disturbances of cellular oxygen homeostasis and their impact on the physiology of body functions (Tretter et al., 2021), as well as oxygen sensing that is being highjacked in cancer (Claesson-Welsh, 2020), are correlated.

## Middle-aging hypovascularity hypoxia hypothesis patterns

### Fundamental cell signaling links

**FIGURE 1:**
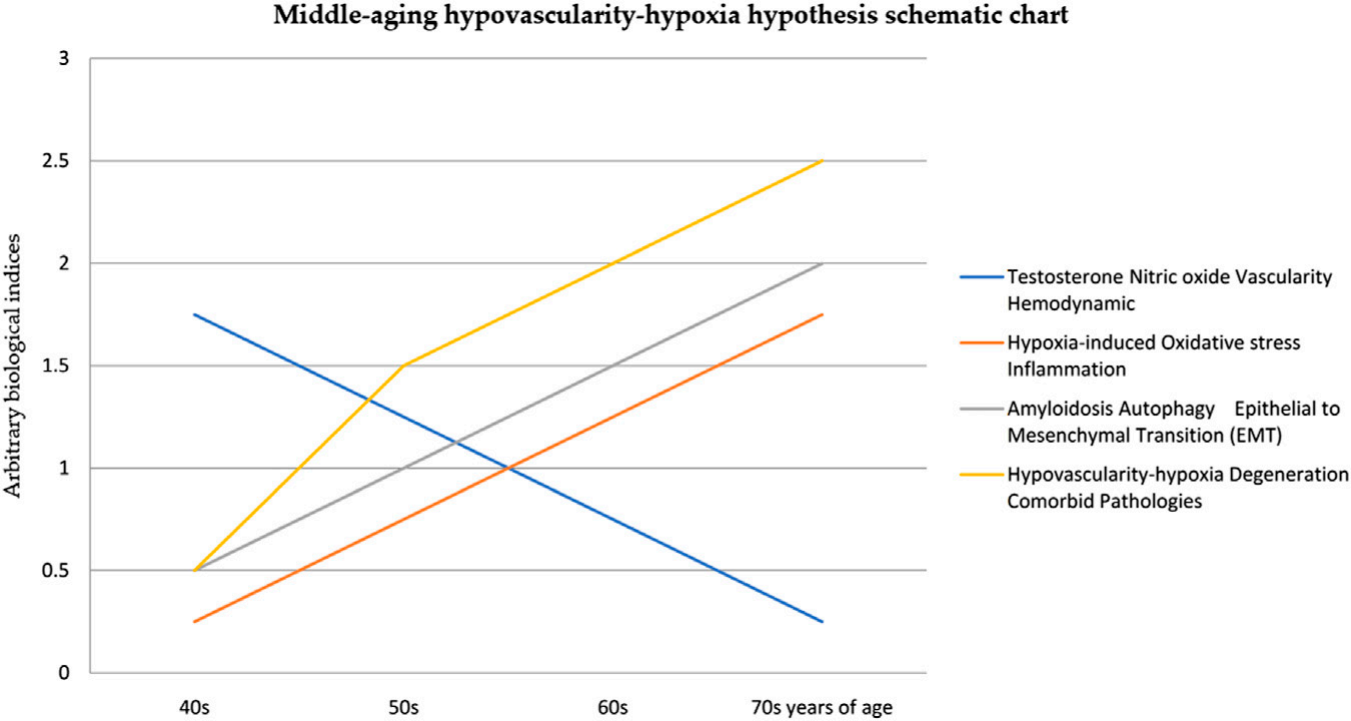
Middle-aging hypoxia hypothesis.

The middle-aging hypovascularity hypoxia hypothesis could provide us with insights into connecting various cell signaling pathways in understanding the etiology of hypoxia genesis and its downstream cellular pathophysiological effects. These are based on the three crucial cell signaling findings that can explain the development of hypovascularity hypoxia (Figure 1):

1. Endocrine signaling: testosterone or estrogen replacement therapy can effectively reverse the testosterone deprivation caused by orchiectomy in rats’ experiments with urethral hypovascularity (Yura et al., 2020; Gerbie et al., 2021), and hypogonadal status patients have been found to have urethral hypovascularity (Hofer and Morey, 2018).
2. Nitric oxide signaling: association of endothelial dysfunction and nitric oxide signaling in the pathogenesis of Alzheimer’s disease (Ahmed et al., 2022) and reduction of estrogen, which lowers nitric oxide bioavailability and induces amyloid deposition, have been observed (Cheboub et al., 2019).
3. Oxygen homeostasis: in a study of aging, the hypoxic response mediated by the hypoxia-inducible factor (HIF) at an environmental hypoxia of 15% oxygen for 6 weeks was associated with higher vascularity and was concluded to be the continuous, non-full-scale activation of the HIF pathway that appears to mediate protection against neurodegeneration (Ollonen et al., 2022). When cells have normal oxygen levels, the HIF is constantly degraded (Jaakkola et al., 2001; Berra et al., 2003; Voit and Sankaran, 2020) through the oxygen-sensing pathway in order to maintain oxygen homeostasis (Yang G. et al., 2020; Claesson-Welsh, 2020; Liao and Zhang, 2020; van Vliet et al., 2021a).

Various aged-related chronic diseases, such as metabolic disorders, cardiovascular diseases, erectile dysfunction, cognition, and cancer, are associated with endocrine (Asih et al., 2017; Diamanti-Kandarakis et al., 2017; Foresta et al., 2017; Cai and Li, 2020; Cannarella et al., 2021; Jockers and Liu, 2021; Leisegang et al., 2021; Assar et al., 2022; Mazzilli et al., 2022; Romejko et al., 2022) and nitric oxide signaling (Radi, 2018; Carlström, 2021; Ledo et al., 2021; Mintz et al., 2021; Pourbagher-Shahri et al., 2021). Systemic microvascular ischemic endothelial dysfunction is a common condition associated with the pathogenesis of diseases (Andersson et al., 2017; Jalnapurkar et al., 2021; Balistreri, 2022), hypoxia (Chen et al., 2008; Jung et al., 2016; Chen et al., 2022a), and vascular remodeling (Yuan and Kevil, 2016; Rajendran P. et al., 2019; Huang et al., 2022). Loss of microvasculature (hypovascularity) implies a developing hypoxic milieu and suggests an important role for chronic hypoxia as an explanation for the progressive nature of fibrosis—the chronic hypoxia hypothesis (Fine and Norman, 2008). Hypoxia is one of the main causes of vascular remodeling (Huang et al., 2022), but it has not been investigated as direct links to the development of hypovascularity hypoxia (declining micro-vessel densities) (Huang and Giordano, 2008).

### Physiological oxygenation perfusion

Oxygen homeostasis and its master regulator, the HIFs, are organizing principles for understanding metazoan evolution, ontology, physiology, and pathology (Semenza, 2010). Aging is accompanied by the development of systemic, gradually increasing hypoxia-related dysfunctions, which are a characteristic of many human diseases (Bowler and Ladomery, 2019; Dzhalilova and Makarova, 2022; Luo et al., 2022). Chronic (continuous, non-interrupted, and sustained) and cyclic (intermittent and transient) hypoxia, which are characterized by fluctuations in oxygen levels (Bader et al., 2020), have been linked to the development of human diseases and cancer (Saxena and Jolly, 2019; Chen et al., 2020; Liu et al., 2022). Chronic sustained hypoxia (CSH) seems to compromise the pulmonary circulation and carotid body stimulation to maintain oxygen levels, whereas the effects of chronic intermittent hypoxia (CIH) appear to be more targeted on the systemic circulation (Prieto-Lloret et al., 2021).

Currently, the experimental cellular oxygen bioavailability levels and findings are confusing and lack clarity in the literature. There are numerous publications showing a wide range of oxygen levels and applications: environmental hypoxia (15% O_2_) (Ollonen et al., 2022), non-physiological hyperoxia (21% O_2_) (Schumacher et al., 2022), normoxia/hypoxia/hyperoxia relativities (Tretter et al., 2020; Tretter, 2022), intermittent hyperoxia–hypoxia paradox (Hadanny and Efrati, 2020), normobaric oxygen paradox (Fratantonio et al., 2021), hyperoxia (100% O_2_)/hypoxia (12% O_2_) (Hommer et al., 2022), normoxia (>8.5% O_2_) (Liu et al., 2022), and “hyperoxic micro-oxygen factories” (Wang W. et al., 2022). This indicates a need to standardize definitions and understand the fluctuations of *in vivo* oxygen bioavailability levels in cellular physiology processes and toxicity (Tessema et al., 2021; Alva et al., 2022; Lius and Syafaah, 2022).

Therefore, due to the lack of standardized definitions, the findings of the hypoxic response mediated by the HIF at “environmental hypoxia” of 15% oxygen for 6 weeks in an aging mice study model, which was associated with higher vascularity (Ollonen et al., 2022) and “physiological hypoxia” at 7% oxygen in culture conditions showing an enhanced microvasculature formation in the laboratory kidney organoid (Schumacher et al., 2022), would need careful interpretation as “physoxia oxygenation perfusion.”

This review focuses on the actual physiological levels of oxygen exposure in normal human tissues *in vivo* (bioavailability) (Alva et al., 2022). A range of about 3%–7.4% oxygen (physoxia)would allow for the comparison of oxygen bioavailability levels between physoxia (5% O_2_), normoxia (20% O_2_), and hypoxia (1% O_2_) (McKeown, 2014). In addition, another term for physoxia is physioxia (Adebayo and Nakshatri, 2022; Alva et al., 2022), which has shown distinct key signaling network expression in laboratory cancer cells to recapitulate their physio-pathological status in the *in vivo* microenvironment (Kumar et al., 2022).

It is the hemodynamic (blood flow) in the microvasculature (microvascular/endothelial) perfusion network (Hesh et al., 2019; Schmid et al., 2019; Taylor and Bordoni, 2022) and not the content of oxygen in the blood that is the main physiological driver of *in vivo* tissue oxygenation perfusion by erythrocytes (Premont et al., 2020). Aging microvasculature (Kalaria and Hase, 2019; Graff et al., 2021) is associated with hypovascularity perfusion, which affects hemodynamics, oxygenation, and vascular remodeling, and is a cause of human diseases (Forsberg et al., 2018; Moeini et al., 2018; Dalby et al., 2019; Santamaría et al., 2020). The primary guarantor of tissue oxygenation is blood flow (hemodynamics) (Jacob et al., 2016), which would be affected by the developing microvasculature hypovascularity.

### Nitric oxide–cyclic guanosine-monophosphate pathway—vascular function

The nitric oxide–cyclic guanosine 3′,5′-monophosphate (NO-cGMP) pathway (Garmaroudi et al., 2016; Mónica et al., 2016; Carlström, 2021) is central for maintaining and sustaining vasodilation (Böger and Hannemann, 2020), vasculature (Costa et al., 2021), and vascular function (Golshiri et al., 2020), as reduced nitric oxide bioavailability can cause endothelial dysfunction (Münzel et al., 2021; Boughaleb et al., 2022), vasoconstriction (Bank et al., 1994; Hannemann and Böger, 2022), and hypoxia (Reinero et al., 2021; Gajecki et al., 2022). Sex hormones, such as testosterone, are linked to the NO-cGMP pathway (Andric et al., 2010), indicating an interdependent relationship between testosterone (androgen) and nitric oxide levels (Hotta et al., 2019; Gur et al., 2020; Zabbarova et al., 2022), which can be related to fluctuations in oxygen perfusion bioavailability (Soni and Padwad, 2017; Nascimento-Filho et al., 2022). The endothelium is an endocrine organ (Stanek et al., 2018; Krüger-Genge et al., 2019) in the human vascular system (Chaudhry et al., 2022), forming the largest microvasculature endothelial surface area network and acting as the gatekeeper of vascular function (Hennigs et al., 2021; Boric and Figueroa, 2022; Howe et al., 2022) in microvasculature cellular communications (Clegg and Mac Gabhann, 2015; Reiterer and Branco, 2020). Such a microvascular dysfunction (endothelial dysfunction) is a common pathophysiological change that occurs in various diseases, such as type 2 diabetes, heart failure, dementia, and depression (Houben et al., 2017; Li W. et al., 2020). This provides a cross-talk between the testosterone–vascular–inflammation-aging triad (Phua, 2021) and nitric oxide signaling (Radi, 2018; Carlström, 2021; Ledo et al., 2021; Mintz et al., 2021; Pourbagher-Shahri et al., 2021).

### Testosterone–vascular–inflamm-aging triad

The testosterone–vascular–inflamm-aging triad (Phua, 2021) is characterized by declining testosterone levels with age over 40 years (Gray et al., 1991; Araujo and Wittert, 2011) and testosterone regulating the NO-cGMP pathway (Andric et al., 2010; Hotta et al., 2019; Gur et al., 2020; Zabbarova et al., 2022). Testosterone deficiency is known to induce endothelial dysfunction (Hotta et al., 2019; Moreau, 2019; Babcock et al., 2022), decrease peri-urethral vascularity (hypovascularity) (Hofer et al., 2017), impair microvascular hyperemia (blood flow) (Corrigan et al., 2015), and reduce nitric oxide production (Vargas et al., 2007; Xiong et al., 2020). In turn, vascular aging is caused by endothelial dysfunction (Donato et al., 2018; Marchio et al., 2019; Rizzoni et al., 2019), which leads to lower peripheral vasodilation (Crecelius et al., 2010; Seals and Alexander, 2018; da Silva et al., 2022), and is correlated with reduced production of nitric oxide (Vanhoutte et al., 2017; Hotta et al., 2019). Inflamm-aging of chronic oxidative stress and inflammation pathophysiology (Phua, 2021) is part of vascular aging (Guzik and Touyz, 2017) and testosterone deficiency (Son et al., 2016; Kataoka et al., 2017; Rovira-Llopis et al., 2017; Babcock et al., 2022).

### Nitric oxide signaling—vasodilation/vasoconstriction physoxia hemodynamics

Healthy tissue function, regulation, and homeostasis are dependent on the vascularity hemodynamic (microcirculation). The vascular endothelium and nitric oxide-mediated signaling govern the regulation of blood microcirculation (Tejero et al., 2019). Nitric oxide bioavailability and expression (Gantner et al., 2020; Akseh et al., 2021; Koukoulis et al., 2022) in signaling transduction (Lundberg and Weitzberg, 2022) through the NO-cGMP pathway (Golshiri et al., 2020) is an important biological aspect for nitric oxide signaling (Radi, 2018; Carlström, 2021; Ledo et al., 2021; Mintz et al., 2021; Pourbagher-Shahri et al., 2021) and the endogenous nitric oxide gasotransmitter (Yang et al., 2016; Nowaczyk et al., 2021) in cancer (Salihi et al., 2022), fibrosis (Chen et al., 2021b), and inflammation (Wang L. et al., 2022). Nitric oxide (NO) acts as a paracrine mediator of vasodilation (Freed and Gutterman, 2017), activating soluble guanylyl cyclase (sGC) in vascular smooth muscle cells and producing cyclic guanosine monophosphate (cGMP). It is this NO-sGC-cGMP signaling pathway that initiates relaxation of the vascular smooth muscle (vasodilation) and inhibits platelet aggregation in both the systemic and pulmonary circulations (Vanhoutte et al., 2017; Böger and Hannemann, 2020). In the systemic circulation, hypoxia results in local vasodilation, which has been shown to be brought about by stabilization of hypoxia-inducible factor-1α (HIF1α) and concomitant upregulation of endothelial nitric oxide synthase (Böger and Hannemann, 2020). In contrast, the physiological response to hypoxia in the pulmonary circulation is vasoconstriction (Böger and Hannemann, 2020). Nitric oxide-mediated activation of cyclic guanosine monophosphate (cGMP) signaling inhibits the acquisition of hypoxia-induced malignant phenotypes in tumor cells (Kim et al., 2020). Nitric oxide deficiency has been associated with the pathophysiological conditions of oxidative stress and inflammation (Shefa et al., 2017; Abdel-Zaher et al., 2021; Bayarri et al., 2021), which is similar to the testosterone–vascular–inflamm-aging triad (Phua, 2021).

Based on these facts, therapeutics that maintain and sustain nitric oxide bioavailability and expression (Gantner et al., 2020; Akseh et al., 2021; Koukoulis et al., 2022) through the NO-mediated cGMP pathway would indicate nitric oxide modulation of oxygen sensing (Berchner-Pfannschmidt et al., 2007; Hickok et al., 2013). Nitric oxide signaling donor/enhancer therapeutics (Andersson, 2018; Yang et al., 2021b; Lundberg and Weitzberg, 2022) would provide vasodilation hemodynamics and physoxia (physiological) oxygenation pharmacodynamics. Conversely, the therapeutics that cause nitric oxide downregulation would provide vasoconstriction hemodynamics and hypoxia pharmacodynamics. Such opposing therapeutic pharmacodynamic treatments showed diametrically opposed biological outcomes and side effects.

### Nitric oxide in oxygen sensing: a new approach and strategy

Henceforth, modulation of nitric oxide in oxygen sensing (Berchner-Pfannschmidt et al., 2007; Hickok et al., 2013) is a new approach and strategy to understand “physoxia” (physiological) oxygenation (Lam et al., 2019; Mas-Bargues et al., 2019; Merkhan et al., 2021; Reiterer et al., 2022) perfusion in physoxia–NO-mediated rejuvenation–regeneration (Hachmo et al., 2020; Kamat et al., 2021; Rando and Jones, 2021) through the blood vasculature (Rodriguez et al., 2021). The physiological oxygen concentration is crucial for culturing stem cells for use in tissue engineering and regenerative medicine (Mas-Bargues et al., 2019), which can reduce the cytokine profiling of the human mesenchymal stem cell secretome (Merkhan et al., 2021). Nitric oxide-mediated vasodilation of vasculature hemodynamics (Freed and Gutterman, 2017; Böger and Hannemann, 2020) would provide necessary physoxia oxygenation perfusion for cells to constantly degrade the HIF (Jaakkola et al., 2001; Berra et al., 2003; Voit and Sankaran, 2020) through the oxygen-sensing pathway in order to maintain oxygen homeostasis (Yang G. et al., 2020; Claesson-Welsh, 2020; Liao and Zhang, 2020; van Vliet et al., 2021b). Therefore, such nitric oxide-enhanced oxygenation hemodynamic mechanisms (Dewhirst et al., 2005; Shu et al., 2015; Adams et al., 2021; Costa et al., 2021) can be seen as physoxia–NO-mediated reduction of hypoxia-induced oxidative stress and inflammation. NO is an important part of the host defense mechanism and play a protective role at the inflammatory site (Iwata et al., 2020).

### Nitric oxide bioavailability and expression signaling

Androgens have been shown to modulate the effects of CIH on the brain (Snyder et al., 2018), correlate with metabolic, vascular, diabetic, and obesity parameters (Groti Antonič et al., 2020) and attenuate hypoxia-induced hypertension (Jiang et al., 2021). Testosterone has been shown to positively regulate functional human corpus cavernosum activities through inhibition of phosphodiesterase type-5 (PDE5) expression and the formation of cGMP and nitric oxide (Gur et al., 2020). Serum levels of testosterone are closely related to levels of endothelial nitric oxide levels (Akseh et al., 2021). It also possesses anti-oxidant (Mancini et al., 2008; Popp Marin et al., 2010; Mendell and MacLusky, 2019; Koukoulis et al., 2022) and anti-inflammatory (Aminuddin et al., 2019; Zhang et al., 2021b; Nasser et al., 2021; Rastrelli et al., 2022) pharmacodynamic properties. Testosterone replacement therapy is used to prevent type 2 diabetes (Haider et al., 2020; Wittert et al., 2021; Yeap and Wittert, 2022), erectile dysfunction (Canguven et al., 2016; Podlasek et al., 2016), and penile fibrosis (Montorsi and Oettel, 2005; Iacono et al., 2012) and to treat lower urinary tract symptoms (Ko et al., 2013; Yassin et al., 2014; Okada et al., 2018).

Testosterone has been shown to inhibit the expression of PDE5 (Gur et al., 2020). PDE5 is part of the NO-sGC-cGMP-PDE5 signaling pathway (Bajraktari et al., 2017; Gur et al., 2020), and its inhibition has been associated with potentiating cancer therapy (Muniyan et al., 2020), counteracting diabetic heart kinetics (Pofi et al., 2022), ameliorating heart failure through cGMP-dependent protein kinase (PKG) activation (Zhu et al., 2022), and supporting increased blood perfusion oxygenation of the vasculature (Giuliano et al., 2013). PDE5 inhibitors restore nitric oxide (Kalsi et al., 2005; Lee et al., 2022) and are selective vasodilators of the NO-cGMP signaling pathway (Aversa et al., 2019; Ahmed et al., 2021). PDE5 inhibitors have been shown to benefit vasculature oxygenation (Morelli et al., 2011; Michel et al., 2015), prevent ischemia–hypoxia (Saito et al., 2014; Zarifpour et al., 2015; Fujii et al., 2019), and reduce microvascular/endothelial dysfunction (Cellek et al., 2014; Ölmestig et al., 2020; Statsenko and Urologiia, 2021); they also reduce oxidative stress (Matsuo et al., 2020), inflammation (Vignozzi et al., 2013; Peixoto and Gomes, 2015), amyloids (Kang et al., 2022), and prostate weight (Kobayashi et al., 2022).

L-arginine/L-citrulline (Shatanawi et al., 2020; Wu G. et al., 2021; Bahadoran et al., 2021; Wijnands et al., 2021) and curcumin (Santos-Parker et al., 2017; Changal et al., 2020; Alidadi et al., 2021; Li K.-X. et al., 2022) are supplements that increase nitric oxide production through their respective L-arginine–nitric oxide pathways (Fleszar et al., 2019; Cziráki et al., 2020; Bahadoran et al., 2021). The increased bioavailability of nitric oxide improves vascular and endothelial function, which is beneficial for human health.

### Anti-androgenic therapeutics

Androgen deprivation therapy (ADT) can cause side effects such as weight gain and emotional changes, and it increases the risk of cardiovascular disease, diabetes, and osteoporosis (MacLennan et al., 2023). The European Association of Urology guidelines recommend not offering neoadjuvant ADT before surgery for patients with prostate cancer (MacLennan et al., 2023). ADT increased markers of oxidative stress/inflammation and the serum levels of thromboxane A2 (TXA2), which is associated with cardiovascular risk (Álvarez-Maestro et al., 2021) and endothelial dysfunction (Gilbert et al., 2013; Teoh et al., 2022), similar to that seen in testosterone deficiency (Hotta et al., 2019; Moreau, 2019; Babcock et al., 2022).

Anti-androgenic therapeutics imply nitric oxide downregulation and are known to cause reductions in hemodynamics (Angrimani et al., 2020; Yoon et al., 2020), microvascular density (hypovascularity) (Hochberg et al., 2002; Donohue et al., 2005; Khwaja et al., 2016; Sun et al., 2018; Khera et al., 2020), and inflammation (Saylor et al., 2012; Hoogland et al., 2021; Nazha and Bilen, 2021). Finasteride-related anti-androgenic therapy is associated with an increased risk of higher-grade prostate cancer (Scailteux et al., 2019; Hu et al., 2020), erectile dysfunction (Fertig et al., 2017), and the post-finasteride syndrome of adverse side effects (Gupta M. A. et al., 2020; Diviccaro et al., 2020; Traish, 2020; Howell et al., 2021; Saengmearnuparp et al., 2021). Additionally, finasteride-related penile curvature/Peyronie’s disease is associated with the most adverse drug reaction reports (Schifano et al., 2022), and this Peyronie’s disease is associated with low testosterone (Askari et al., 2019), inflammation (Patel et al., 2020; Swislocki and Eisenberg, 2021), and fibrosis of the tunica albuginea (Chung et al., 2020; Segundo and Glina, 2020).

### Translational imaging technologies and retinal microvasculature

Hypovascularity (Hofer and Morey, 2018; Yura et al., 2020; Gerbie et al., 2021) is the detectable loss of micro-vascularity, a geometric structure that serves as an interface hub for hemodynamics, oxygenation, and perfusion exchanges (Secomb, 2016; Pollock et al., 2022). Currently, the role of translational imaging technologies in interrogating the structural and functional status of the microcirculation for clinical applications in human diseases is being explored (Guerraty et al., 2021). There have been numerous retinal microvascular structural and blood flow studies that have shown an association with vascular aging (Wei et al., 2017; Orlov et al., 2019; Arnould et al., 2022; Gómez-Sánchez et al., 2022), sex hormones (Nuzzi et al., 2018; Feng et al., 2021; Aribas et al., 2022), and chronic diseases such as neurodegeneration/diabetic retinopathy (Marques et al., 2022), coronary artery disease (Zhong et al., 2022), type 2 diabetes (Kim H. et al., 2022), and hypertension (Zeng et al., 2022). Retinal microvasculature structures also responded to oxygen availability (Hommer et al., 2022), an aging reduction at >50 years (Abay et al., 2022), and to the anti-androgenic 5α-reductase inhibitor (Shin et al., 2020).

## Discussion

### Middle-aging hypovascularity hypoxia hypothesis: cyclic/chronic hypoxic milieu

The middle-aging hypovascularity hypoxia hypothesis postulates that the consequences of middle-aging concomitant declining sex hormones (andropause/menopause) and nitric oxide signaling (Phua, 2021) lead to the steady loss of vascularity, hemodynamic changes, and an increasing cyclic/chronic hypoxic milieu (Fine and Norman, 2008) (Figure 1).

Loss of micro-vascularity (Hofer and Morey, 2018; Gerbie et al., 2021) implies an important role for cyclic/chronic hypoxic milieu (Fine and Norman, 2008) in regionalizing perfusion and contributing to the progression of age-related degenerative comorbid diseases in humans. Age-related declining sex hormones (andropause/menopause) are mediated by NO–oxygen sensing (Berchner-Pfannschmidt et al., 2007; Hickok et al., 2013), causing a hypoxic stress condition and dysregulation of the cellular biology of amyloidosis (Cheboub et al., 2019; Ahmed et al., 2022), which is counteracted by autophagy (Chuang et al., 2018; Wang and Le, 2019; Wang and Zhang, 2019), along with the epithelial-to-mesenchymal transition (EMT) (Byrne et al., 2016; Kim I. et al., 2022; Ribatti, 2022; Mohamed et al., 2023). A regionally restricted oxygen perfusion is a cyclic/chronic hypoxic environment that can lead to degenerative pathologies such as hypertrophy, atrophy, fibrosis, and neoplasm due to developing localized hypoxia (Figure 1). Examples of such a range of hypoxic degenerative pathologies can be seen in acute high-altitude hypoxia (hypobaric hypoxia) organ hypertrophy (Pena et al., 2022) and tissue edema (Mesentier-Louro et al., 2021). Others include androgen deprivation therapy promoting epithelial–mesenchymal transition (Byrne et al., 2016), cancer-associated fibroblasts (Kim I. et al., 2022), and prostate cancer progression metastasis (Mohamed et al., 2023).

### Nitric oxide-mediated hypovascularity: hypoxia-dependent processes

**FIGURE 2:**
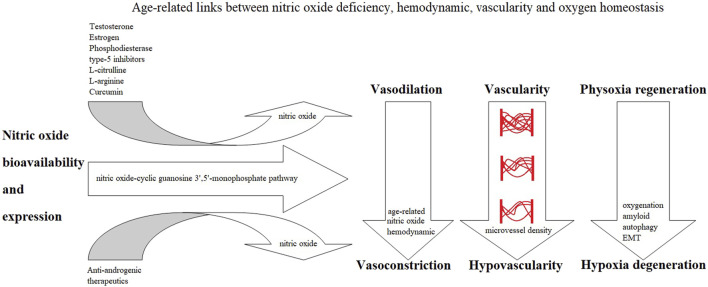
Age-related links.

The prevailing narratives for prostate carcinogenesis progression are typically driven by an androgen-dependent process (Aurilio et al., 2020). However, the hypovascularity hypoxia hypothesis, which has accumulated evidence-based patterns, demonstrates an “androgen-induced nitric oxide-mediated hypovascularity hypoxia-dependent process.” Androgen (hormonal) and hypoxia signaling pathways are separate and independent (Tran et al., 2020). Nitric oxide (Dent et al., 2021) through cGMP (Friebe et al., 2020) provides the fundamental link to various NO-sGC-cGMP (Vanhoutte et al., 2017; Böger and Hannemann, 2020) and NO-sGC-cGMP-PDE5 (Bajraktari et al., 2017; Gur et al., 2020) cell signaling pathways to affect NO-mediated oxygen sensing (Berchner-Pfannschmidt et al., 2007; Hickok et al., 2013), affecting vascularity, hemodynamics, and oxygenation perfusion (Amdahl et al., 2019; Kapil et al., 2020) (Figure 2).

Adult prostate demonstrates remarkable regenerative capacity over multiple cycles of castration and androgen administration, suggesting the existence of an androgen-independent epithelial progenitor in benign prostatic hyperplasia and prostate cancer (Joseph et al., 2021). Androgen and estrogen receptors have been shown to intersect with the HIF/NF-kB signaling in prostate cancer (Russo et al., 2016), and hypoxia increases androgen receptor activity (Park et al., 2006) within a low-androgen environment (Mitani et al., 2011). Higher aggressiveness of prostate cancer correlates with testosterone deficiency (Neuzillet et al., 2019), and the progression of hormone-naïve prostate carcinomas correlates with a low number of vascular vessels (Smentoch et al., 2019). In a case study, superb microvascular imaging (SMI) identified poor internal blood flow in prostate stromal sarcoma (Ohashi et al., 2022).

### Vascular aging hypovascularity niches

Oxygen plays a key role in cellular homeostasis, and physiological oxygen levels in various organs range between 2% and 9% *in vivo*, with the highest levels of 9% in the kidneys and the lowest of 0.5% in parts of the brain (Adebayo and Nakshatri, 2022). Hypovascularity (Yura et al., 2020; Gerbie et al., 2021), reduced microvascular density (Querfeld et al., 2020), and the partially preserved aging microvasculature (Lam et al., 2019) are evidence of decreasing vascularity, hemodynamic perfusion, and physiological oxygenation. Vascular aging, characterized by structural and functional alterations of the vascular wall, is a hallmark of aging (Scioli et al., 2014; Xu et al., 2017; Gkaliagkousi et al., 2022), and vascular endothelial cells can reshape their microenvironment, forming a “niche” (Lei et al., 2022). Decreased blood vessel density and endothelial cell subset dynamics occur during the aging of the endocrine system (J. Chen et al., 2021). In a mouse model of aging, the researchers used super-resolution ultrasound localization microscopy (ULM) and found significant decreases in blood velocity and significant increases in vascular tortuosity across all brain regions in the aged cohort (Lowerison et al., 2022).

**FIGURE 3:**
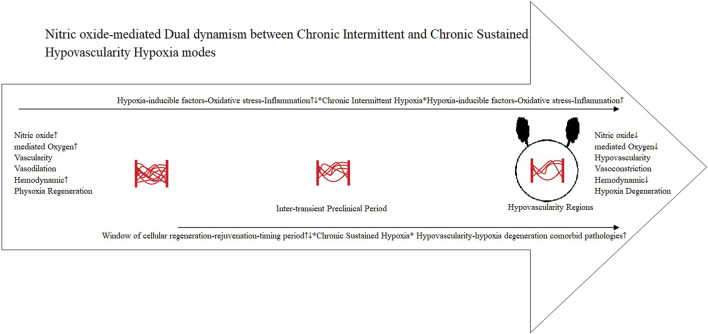
Dual dynamism CIH–CSH.

Such intimate vascular aging relationships are formed during the inter-transient preclinical (Krishnan et al., 2018; Younes et al., 2019; Adebayo and Nakshatri, 2022) period between the physoxia regeneration–hypoxia degeneration and adaptive (Pomatto et al., 2019a; Pomatto et al., 2019b) pathological (Lasne et al., 2006; Gilmore et al., 2021) homeostasis, which is time-dependent (Ming et al., 2013; Byrne et al., 2016; Rouverdo et al., 2023). The dual dynamics of observed CIH and CSH (Böger and Hannemann, 2020; Prieto-Lloret et al., 2021) form integration (local) and extension (region) between these two operating modes through the receding microvasculature network of interactions within the regions of cyclic/chronic hypoxia milieu in the tissues, glands, and organs niche (Minami et al., 2019; Jambusaria et al., 2020; Gifre-Renom et al., 2022; Xu et al., 2022) (Figure 3).

The impaired tissue oxygen delivery is a major cause of organ damage and failure in critically ill patients, even when systemic parameters, including cardiac output and arterial hemoglobin saturation, are close to normal (Roy and Secomb, 2021). Assessments of microvascular function in organ systems are, therefore, crucial (Ku et al., 2021; Xu C. et al., 2022). The median microvascular density was reduced by 29% in skeletal muscle and 24% in the heart in animal models of chronic kidney disease and by 32% in human biopsy, autopsy, and imaging studies (Querfeld et al., 2020). Such a developing cyclic/chronic hypoxia milieu provided the chronic pathological trajectories in rheumatoid arthritis (Fearon et al., 2016; Sabi et al., 2022), cardiovascular (Li et al., 2020b; Godo et al., 2021; Cornuault et al., 2022), and diabetes (Catrina and Zheng, 2021) disorders.

### Hypoxia pathological microenvironment niches

Earlier inter-transient preclinical period vascularity in the CIH-systemic circulation (Böger and Hannemann, 2020; Prieto-Lloret et al., 2021) could adequately respond to physoxia regeneration–rejuvenation (Hachmo et al., 2020; Kamat et al., 2021; Rando and Jones, 2021). However, localized cyclic/intermittent hypoxia (CIH) within a region is a potent proinflammatory stimulus in human diseases (Schaefer et al., 2017; Wilson et al., 2018; Korbecki et al., 2021). Thus, advancing the establishment of a late-stage dysfunctional vasculature through vascular remodeling (receding) (Ouarné et al., 2021; Ahmed et al., 2022; Ollonen et al., 2022), which would extend the “CSH compromised pulmonary circulation” region (Böger and Hannemann, 2020; Prieto-Lloret et al., 2021) into an integrated, developing, localized CSH self-perpetuating hypovascularity cyclic/chronic hypoxia pathological microenvironment niche (Carnero and Lleonart, 2016; Macharia et al., 2021; Frisbie et al., 2022) (Figure 3).

Pathological processes associated with hypoxia in age-related diseases are increasingly being recognized in chronic liver disease (Wan et al., 2022), chronic kidney disease (Wang B. et al., 2022), lower urinary tract symptoms—diabetes (Abler and Vezina, 2018), chronic prostatic disease (Luisetto et al., 2019), and prostate carcinogenesis (Deep and Panigrahia, 2015; Ashton and Bristow, 2020; Mohamed et al., 2023). Cyclic/intermittent hypoxia (CIH) induces a replication catastrophe, resulting in an increase in the activity of APOBEC3B (Bader et al., 2021). Hypoxia induces genomic instability through an increase in mutation frequency and enhanced replication stress, inhibiting DNA repair (Hassan Venkatesh et al., 2020; Kaplan and Glazer, 2020; Saitoh and Oda, 2021). Tumor initiation and progression are somatic evolutionary processes driven by the accumulation of genetic alterations (Lahouel et al., 2020; Zahir et al., 2020), and timing analyses suggest that driver mutations often precede diagnosis (preclinical) by many years, if not decades (Gerstung et al., 2020).

### Window of cellular regeneration–rejuvenation: window of opportunity and timing hypothesis

Taken together, the critical inter-transient preclinical period (disease/cancer) of physoxia–NO-mediated hemodynamic (Lam et al., 2019; Mas-Bargues et al., 2019; Merkhan et al., 2021; Reiterer et al., 2022) window of cellular regeneration–rejuvenation (Hachmo et al., 2020; Kamat et al., 2021; Rando and Jones, 2021) can be associated with the window of opportunity and timing hypothesis (Figure 3). The window of opportunity and timing hypothesis is the critical timing of hormone therapy initiation with respect to age and/or the menopause (and andropause) transition, and optimal effects are evident with early initiation (Maki, 2013; Speth et al., 2018).

Current observational data show that menopause/hormonal replacement therapy (MPT/HRT) reduces “all-cause mortality” (Akter et al., 2022; Bluming, 2022; Hodis and Mack, 2022; The North American Menopause Society, 2022), decreases the risk of dementia among female patients with depression (Kim T.-Y. et al., 2022), lowers the risk of breast cancer (in Korea) (Baek et al., 2022), and reduces COVID-19 deaths (Sund et al., 2022). Testosterone replacement therapy (TRT) is recommended for late-onset hypogonadism in aging males over 65 years old (Nieschlag, 2020; Burte et al., 2021), but it is still a subject of controversy (Diokno, 2022; Mian et al., 2022). Regardless of testosterone levels, most symptomatic late-onset hypogonadism has been shown not to be correlated (Li et al., 2020c; Ishikawa et al., 2020; La Vignera et al., 2020; Tsuru et al., 2022).

Findings from a randomized clinical trial of testosterone therapy showed that it does not affect lower urinary tract symptoms, but it does improve markers of prostatitis in men with benign prostatic hyperplasia (Rastrelli et al., 2022). This can be reflective of the dual dynamism between the CIH and CSH modes (Böger and Hannemann, 2020; Prieto-Lloret et al., 2021), in which testosterone therapy is not effective on the lower urinary tract symptoms of the already established hypoxic pathological symptomatic localized CSH niche. Conversely, the partially preserved aging microvasculature would still provide the pro-vasculogenic CIH systemic effects (Lam et al., 2019) of testosterone therapy to improve markers of prostatitis in men with benign prostatic hyperplasia (Rastrelli et al., 2022) (Figure 3).

### Epithelial–mesenchymal transition

EMT encompasses dynamic changes in cellular organization from epithelial-to-mesenchymal phenotypes, which leads to functional changes in cell migration and invasion, in a diverse range of physiological and pathological conditions (Yang J. et al., 2020). These changes occur during embryogenesis (type 1 EMT), wound healing, tissue regeneration, fibrosis (type 2 EMT) and in cancer, where they contribute to cell stemness (plasticity), drug resistance, immune escape, and metastasis (type 3 EMT) (Manfioletti and Fedele, 2022). EMT can be seen as a period of a bet-hedging—evolutionary cellular adaptation (Jolly et al., 2018; Capp and Thomas, 2022; Jain et al., 2022), which diversifies mesenchymal phenotypes (cancer stemness) and increases their survival (Mortezaee and Majidpoor, 2022) in changing hypoxia-induced pathophysiology (Lee et al., 2020; Papale et al., 2020; Nushtaeva et al., 2023; Zhang et al., 2023).

Hypoxia promotes the aggressiveness of prostate cancer by upregulating the expression of the EMT activator Zeb1 and SK3 channel (Bery et al., 2020). HIF-α promotes the migration and invasion of cancer-associated fibroblasts by miR-210 (Yang Q. et al., 2021). EMT regulators Twist, Slug, and Snail are associated with poor prostate cancer prognosis (Børretzen et al., 2021) and the transforming growth factor-β1 (TGF-β1) cytokine in the tumor microenvironment with autophagy induction (Jena et al., 2022).

Genetically modified mouse experimental models with enrichment of luminal progenitor cells in prostate inflammation, benign prostate hypertrophy, and prostate cancer and the intrinsic castration tolerance of these cells in resistance to androgen deprivation therapy suggest a role in carcinogenesis (Baures et al., 2022). Genomic analysis of benign prostatic hyperplasia implicates cellular re-landscaping in disease pathogenesis (Middleton et al., 2019), and 5-alpha reductase inhibitors (anti-androgenic) induce a prostate luminal to club cell transition in human benign prostatic hyperplasia (Joseph et al., 2022).

### Mesenchymal-to-epithelial transition

EMT is reversal by the mesenchymal-to-epithelial transition (MET) (Yang J. et al., 2020; Manfioletti and Fedele, 2022), which can be synonymous to type 2 EMT, a reparative-associated fibrotic process in response to chronic inflammation (Marconi et al., 2021). The chief candidate for EMT reversal (regeneration/rejuvenation) would be the timely amelioration of hypoxia (López-Novoa and Nieto, 2009; Lee et al., 2020; Papale et al., 2020; Nushtaeva et al., 2023; Zhang et al., 2023) before the establishment of dysregulation–degeneration hypoxic cell biology (Phua, 2021; Rando and Jones, 2021) and cellular senescence (Welford and Giaccia, 2011; Barabutis et al., 2018; Lacroix et al., 2020; Otero-Albiol and Carnero, 2021). In a review article, new approaches to alleviating hypoxia through the modulation of the vascular state in the tumor microenvironment offer promise for ovarian cancer immunotherapeutic strategies (Klemba et al., 2020). Therefore, the reversal from EMT to MET can also be associated with the critical inter-transient preclinical (disease/cancer) period of time-dependent (Ming et al., 2013; Byrne et al., 2016; Rouverdo et al., 2023) physoxia–NO-mediated hemodynamic (Lam et al., 2019; Mas-Bargues et al., 2019; Merkhan et al., 2021; Reiterer et al., 2022) window of cellular regeneration–rejuvenation (Hachmo et al., 2020; Kamat et al., 2021; Rando and Jones, 2021) and the window of opportunity and timing hypothesis (Maki, 2013; Speth et al., 2018).

*In vitro* regenerative medicine experiments can provide evidence to support the favorable benefits of physoxia/physioxia oxygenation (Yttersian Sletta et al., 2017; Dennis et al., 2020; Dougherty et al., 2020; Dogan et al., 2022), growth factors (Wang et al., 2007; Zhang et al., 2021a; Kataoka et al., 2022), androgen (Bui et al., 2017; Nyquist et al., 2019), inflammation reduction (Shephard et al., 2022), and enhanced microvasculature (Schumacher et al., 2022). Aging reprograms the hematopoietic vascular niche to impede regeneration and promote fibrosis (Chen et al., 2021c) within blood vessel wall-associated tissue remodeling (Craig et al., 2022), and it also play a role in the association of cardiac fibroblasts and endothelial cells in myocarditis (Xuan et al., 2022).

### Cellular senescence—autophagy

Cellular senescence persists during aging and promotes age-related pathologies through the pro-inflammatory senescence-associated secretory phenotype (SASP) (Covarrubias et al., 2020; van Vliet et al., 2021b), and its expression is dependent on oxygenation levels (van Vliet et al., 2021b). The contemporary aspects of age-related cellular senescence pathologies can also be the part of a crosstalk within the geroscience perspective in the characterization of the SASP as the “remodeling-associated secretory phenotype” (Gems and Kern, 2022), mechanisms of vascular aging (Ungvari et al., 2020), and in the role of aging endocrine diseases (Khosla et al., 2020).

The early protective role of wild-type p53 in suppressing inflammation and cancer is strongly associated with the regulation of important cellular activities of the cell cycle of senescence and apoptosis (Barabutis et al., 2018; Agupitan et al., 2020; Lacroix et al., 2020). The missense mutations in the TP53 gene are found most frequently across all cancer types and give rise to mutant p53 proteins that lose their tumor suppressive activities (Mantovani et al., 2019; Sabapathy and Lane, 2019; Stein et al., 2019).

Cellular senescence is a specialized form of growth arrest and plays a critical role in tumor suppression and aging, with autophagy being activated during the process of senescence (Yang et al., 2012; Rajendran S. et al., 2019; Tabibzadeh, 2023). Oxygen concentration can modulate cellular senescence and autophagy in human trophoblast cells (Seno et al., 2018).

Autophagy plays a role in early tumor suppression in terms of the cell regulation pathways and in their dysregulation in late stages, where they act as tumor promoters (Dower et al., 2018; Alvarez-Meythaler et al., 2020; Li H. et al., 2020; Lim and Murthy, 2020). Impaired autophagy predisposes individuals to age-related diseases, whereas interventions that stimulate autophagy often promote longevity (Leidal et al., 2018; Condello et al., 2019; Luo and Qin, 2019; Wong et al., 2020).

### Penile rehabilitation oxygenation

Indeed, such a “physoxia-mediated” (oxygenation) hemodynamic is used for the early therapeutic effect of penile rehabilitation after prostatectomy (Marcu et al., 2020; Osadchiy et al., 2020; Nicolai et al., 2021). Regular erections (Montorsi and Oettel, 2005) are used to improve oxygenation and hemodynamics (reducing hypoxia and inflammation) (Welliver et al., 2014; Hadanny et al., 2018; Sen et al., 2020) and preserve the endothelial structure using PDE5 inhibitors (Elkamshoushi et al., 2021) to prevent penile fibrosis (El-Sakka and Yassin, 2010; Kaminsky et al., 2011). Long-term testosterone therapy improves long-term blood circulation of penile arteries, penile length and girth, erectile function, and nocturnal penile tumescence and duration (Canguven et al., 2016). Electrical penile erection stimulation in mice induced angiogenesis, cell survival, proliferation, and anti-fibrosis signaling pathways (Kwon et al., 2016).

### Hypoxia-related carcinogenesis and chronic diseases

Both oxidative stress and inflammation are driven by hypoxia (Biddlestone et al., 2015; McGarry et al., 2018; Korbecki et al., 2021; Mesentier-Louro et al., 2021), which could explain why anti-oxidative therapies alone cannot restore cellular redox homeostasis (Tretter et al., 2021). Endothelial cell biology of functions and dysfunctions (Charreau, 2022) is an emerging approach to understanding microvascular endothelial heterogeneity and inflammation (Yang et al., 2021c; Rossi et al., 2022). Impaired endothelial function is thought to contribute to the increased cardiovascular risk (Craighead et al., 2020), vascular aging associated with atherosclerotic ischemic stroke (Koutsaliaris et al., 2022), and oxidative stress–inflammation in chronic kidney disease (Ebert et al., 2021). Aging is associated with chronic low-grade inflammation, cancer incidence, and mortality (Guerville et al., 2022), a physiological process mediated by numerous biological and genetic pathways, which are a driving force for all age-related diseases (Li et al., 2021). Cancer often arises in the context of an altered tissue microenvironment landscape (Laconi et al., 2021).

This pathophysiology of oxidative stress–inflammation induced by hypoxia is a response to humans’ exposure to acute high-altitude hypoxia (Malacrida et al., 2019; Mrakic-Sposta et al., 2022; Pham et al., 2022). Nature’s adaptive responses to nitric oxide emphasize the importance of nitric oxide’s vasodilator role in the native inhabitants living in high-altitude hypoxia environments (Qi-sheng et al., 2021; James et al., 2022). Genetically similar East African highlanders, the Amhara tribe, balance minimally elevated hemoglobin with a vasodilatory response to environmental hypoxia, whereas the Oromo tribe mainly relies on an elevated hemoglobin response (Cheong et al., 2017; Getu, 2022). Newborn llamas from the highland region have a reduced pulmonary vasoconstriction response to acute hypoxia due to an enhancement of NO pathways (Reyes et al., 2018). There is an association between 17β-estradiol receptors and nitric oxide signaling that augments the high-altitude adaptation of Ladakhi highlanders (Pooja et al., 2021). Lower mortality rates from cardiovascular diseases, diabetes, and cancers are seen in native highland residents (Thiersch and Swenson, 2018; Wander et al., 2020; Burtscher et al., 2021).

Hypoxia plays a critical role in shaping the genomic and evolutionary landscapes of cancer (Bhandari et al., 2020; Zhang X. et al., 2020), with a multifaceted interplay between hormones, growth factors, and hypoxia in a tumor microenvironment milieu (Lappano et al., 2022), including the HIFs (Satija et al., 2021; Sebestyén et al., 2021) and transforming growth factor (TGF-β) produced in the hypoxic, chronic inflammatory settings (Mortezaee and Majidpoor, 2022). Androgen deficiency/deprivation caused drastic endothelial dysfunction, resulting in reduced blood flow (ischemia/hypovascularity) to the prostate gland (Angrimani et al., 2020; Jin Cho and Pyo, 2020; Yoon et al., 2020), causing an ischemia–hypoxia stress tissue microenvironment (Thurmond et al., 2015; Byrne et al., 2016). Hypoxia stress caused the induction of amyloidosis-autophagy-EMT cell signaling interactions, beginning with amyloidosis (Cheboub et al., 2019; Phua, 2021) to allow cells to enter a dormant/resting stage (Audas et al., 2016; Mizejewski, 2017; Pavliukeviciene et al., 2019). Age-related amyloidoses (Rubel et al., 2020; Tasaki et al., 2021) are increasingly being discussed in the mainstream literature, including a range of organs: brain/Alzheimer’s (Shea et al., 2019; Schweighauser et al., 2022), renal amyloidosis (Gupta N. et al., 2020; Herrera, 2021; Gurung and Li, 2022), eyes/Alzheimer’s retinopathy (Mirzaei et al., 2020), type 2 diabetes/Alzheimer’s (Wang and Westermark, 2021), and cardiac amyloidosis (Garcia-Pavia et al., 2021; Hänselmann et al., 2022).

In turn, the amyloid protein aggregates are countered by autophagy and the ubiquitin proteasome system (Chuang et al., 2018; Wang and Le, 2019; Wang and Zhang, 2019). The availability of the HIFs in the hypoxia and inflammation pathophysiological states is primarily regulated post-translationally through the ubiquitin proteasome system (autophagy) (Günter et al., 2017; Cohen et al., 2019). HIFs enable cells to adapt to decreased oxygen bioavailability (Albanese et al., 2020; Hirota, 2020) with stochastic fluctuations of oxygen that will select for the bet-hedging (Warburg) phenotype (EMT) (Gravenmier et al., 2018). Under conditions of hypoxia, most eukaryotic cells can shift their primary metabolic strategy from oxidative phosphorylation to increased aerobic glycolysis, known as the Warburg effect (Elzakra and Kim, 2021; Kierans and Taylor, 2021).

Hypoxia in chronic kidney disease (Faivre et al., 2021; Wang B. et al., 2022) has been well studied and is used here to highlight the middle-aged (>50 years) (Ryu et al., 2019) hypovascularity (Fine and Norman, 2008; Evans et al., 2020; Querfeld et al., 2020) hypoxia (Faivre et al., 2021; Wang L. et al., 2022) pathological trajectories in linking the endocrine system (Zhao and Schooling, 2020; Li L. et al., 2022; Romejko et al., 2022), nitric oxide signaling, and oxygen homeostasis pathophysiology (Burmakin et al., 2021; Carlström, 2021; Xu M. et al., 2022; Edwards and Kurtcuoglu, 2022). Oxidative stress–inflammation pathophysiology (Ebert et al., 2021), amyloidosis (Ryu et al., 2019; Herrera, 2021; Gurung and Li, 2022), autophagy (Tang et al., 2020), and EMT (Chen et al., 2022b) would result in degenerative hypertrophy, fibrosis, atrophy, and neoplasm (Ryu et al., 2019; Pinto et al., 2021) comorbid conditions (Charles and Ferris, 2020). Moreover, PDE5 inhibitors can have beneficial renal protective effects by improving hemodynamics and reducing oxidative stress and inflammation (Georgiadis et al., 2020; Coskuner and Ozkan, 2021).

### Middle-aging sex hormone—endogenous nitric oxide-mediated oxygen homeostasis

Maintenance of cellular oxygen homeostasis during sex hormone–nitric oxide downregulation and hemodynamic reduction due to hypovascularity is the key physiological challenge during middle age (Kumar and Choi, 2015; Gravenmier et al., 2018; Janaszak-Jasiecka et al., 2021; Assar et al., 2022; Xu C. et al., 2022). When “physoxia” oxygen delivery is disrupted by microvascular hypovascularity (ischemia–hypoxia), it triggers intrinsic adaptive biological processes to facilitate heterogeneous cell survival in the hypoxia-degenerative environment (Feil et al., 2022; Wicks and Semenza, 2022; Yang et al., 2022). This indicates a critical juncture of timing when this window of “cellular regeneration–rejuvenation–timing” is made possible when oxygen levels are restored to normal “physoxia” conditions (Dogan et al., 2021, 2022; Merkhan et al., 2021).

The aim of early intervention is to prevent physiological oxygen deprivation (hypoxia) related to aging (Fine and Norman, 2008; Kumar and Choi, 2015; Claesson-Welsh, 2020; Wei et al., 2022). Androgens have the potential to prevent age-related impairment in ischemia-induced neovascularization (Lam et al., 2019; Gerbie et al., 2021) and could provide hemodynamic physoxia oxygenation to continually act on HIF degradation through the oxygen-sensing pathway (Jaakkola et al., 2001; Berra et al., 2003; Strowitzki et al., 2019; Voit and Sankaran, 2020).

Human nitric oxide production decreases with age, losing 50% by age 40 and 85% by age 65 (Gerhard et al., 1996; Taddei et al., 2001). This preclinical period coincides with the science of nitric oxide in all chronic diseases being associated with decreased blood flow to the affected organ, resulting in increased inflammation, oxidative stress, and immune dysfunction (Bryan et al., 2023). Lack of nitric oxide bioavailability in post-menopausal women is well documented (Novensà et al., 2011; Fredette et al., 2018; Somani et al., 2019), and menopause/hormonal replacement therapy (MPT/HRT) can replace nitric oxide (Best et al., 1998; Cicinelli et al., 1999; Bednarek-Tupikowska et al., 2008). The estrogen replacement therapy has been shown to reduce oxidative stress (Bellanti et al., 2013; Unfer et al., 2015; Borrás et al., 2021), reduce oxidative stress/inflammation (Vural et al., 2006; Georgiadou and Sbarouni, 2009; Jee et al., 2021; Estrada-Cruz et al., 2022), and improve hemodynamics (Redberg et al., 2000; Light et al., 2001; Deschênes et al., 2010). Similarly, testosterone replacement therapy can reduce oxidative stress (Mancini et al., 2008; Popp Marin et al., 2010; Mendell and MacLusky, 2019; Koukoulis et al., 2022) and inflammation (Bianchi, 2019; Mohamad et al., 2019; Rastrelli et al., 2019) and improve hemodynamics (Efesoy et al., 2018; König et al., 2019; Cipriani et al., 2021). This indicates that sex hormones can reduce hypoxia-induced oxidative stress and inflammation during the critical window of cellular regeneration, rejuvenation, and timing period through physoxia–NO-mediated hemodynamic oxygenation. In cancer, the two-concentration (biphasic) hypothesis of nitric oxide has determined that low levels of nitric oxide are cancer promoting, while high levels of nitric oxide are protective against cancer (Soni et al., 2020).

Comorbidities in the middle-aged group (35–59 years) of ischemic heart disease were less severe than those of the older age group (60–69 years) (Zhou et al., 2022), with the first chronic condition developing in the 50s or 60s (Zhu et al., 2018). Data analysis showed a reduction in cardiovascular disease and breast cancer in women aged under 60 years who were on hormone replacement therapy, as seen in the Women’s Health Initiative Trial (Langer, 2017; Lobo, 2017; El Khoudary et al., 2020). Women who were BRCA1/BRCA2 mutation carriers and were under 45 years of age and who received risk-reducing salpingo-oophorectomy and hormonal replacement treatment did not affect their breast cancer rates (Michaelson-Cohen et al., 2021). Furthermore, premenopausal women are better protected against cardiac hypertrophy (Wu et al., 2020). Sleep quality and the accumulation of cortical amyloid-β are associated in post-menopausal women from the Kronos Early Estrogen Prevention Study (Zeydan et al., 2021) and the long preclinical period (approx. 10–15 years) prior to symptomatic Alzheimer disease onset (Younes et al., 2019; Elman et al., 2020; Elman-Shina and Efrati, 2022).

Prostate cancer incidence is rare for those under 50 years of age, increasing to 1 in 52 by age 59 and to more than 1 in 2 at age 65 or older (Rosario and Rosario, 2022). Higher testosterone levels are associated with smaller prostate size (Xia et al., 2021), and younger age testosterone replacement therapy leads to prostate stabilization (Zhang Q. et al., 2020). In prostate cancer patients, low serum testosterone has been found to be associated with androgen receptor expression (Schatzl et al., 2002; Husain et al., 2016; Feng and He, 2019; Hashmi et al., 2019).

### Nitric oxide physiology and the nitrate–nitrite–nitric oxide pathway

Nitric oxide is a strong vasodilatory and anti-inflammatory signaling molecule that plays diverse roles in maintaining vascular homeostasis (Cyr et al., 2020), biological functions (Gantner et al., 2020), and in carcinogenesis (Mintz et al., 2021). This new strategy, as a physoxia–NO-mediated mechanism, allows for a causal relationship (Kiani et al., 2022) to be understood in the aftermath of sex hormone–nitric oxide downregulation with the non-canonical pathways for nitric oxide synthesis in the body, known as the nitrate–nitrite–nitric oxide pathway (Kapil et al., 2020; Mintz et al., 2021).

Findings are consistent with observational reports linking dietary nitric oxide sources to beneficial health outcomes associated with the Mediterranean diet (Martínez-González et al., 2015; Shannon et al., 2021), dietary spermidine (Wu et al., 2022), and longevity (healthspan) in Blue Zone populations (Vasto et al., 2014; Nieddu et al., 2020). The Mediterranean diet increases serum nitric oxide (Shannon et al., 2018; Mohajeri and Cicero, 2023), improves endothelial function (Shannon et al., 2020; Fatima et al., 2023), is anti-oxidant (Gantenbein and Kanaka-Gantenbein, 2021; Karbasi et al., 2023), and is anti-inflammatory (Tsigalou et al., 2020; Soda et al., 2021). Beetroot juice as a dietary nitrate supplementation improves peripheral blood flow, endothelial function, and anti-inflammatory status in individuals with Raynaud’s phenomenon (Shepherd et al., 2019).

## Conclusion

Both these hypotheses—the prostate aging degeneration (Phua, 2021) and middle-aging hypovascularity hypoxia—provide complimentary evidence supporting the importance of time-dependent maintenance of vascular function and vascularity hemodynamics, respectively.

**FIGURE 4:**
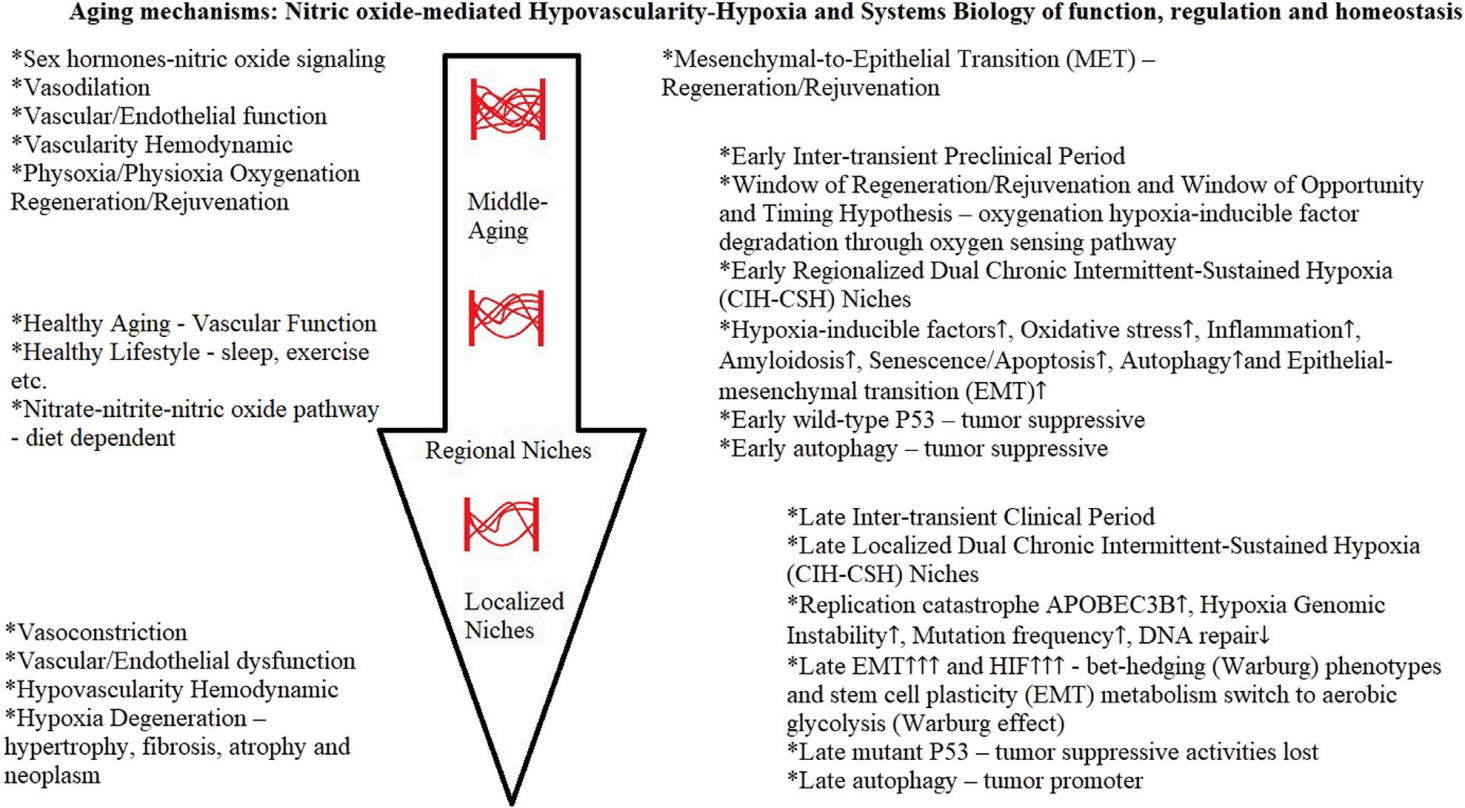
Aging mechanisms.

In a Mendelian randomization analysis study, the evidence suggested that menopause accelerates the epigenetic aging of blood (Levine et al., 2016). Hypoxia is one of the common characteristics of cancer (Liao et al., 2023), and the hypovascularity hypoxia hypothesis provided evidence of an early hypoxia milieu genesis during middle-age nitric oxide-mediated vascular aging. Cancer hypoxia is one of the most important hallmarks of cancer; it affects gene expression, metabolism, and ultimately, tumor biology-related processes (Sebestyén et al., 2021). All in all, this scoping review can provide the clarity of concepts and patterns to determine the aging mechanisms as a consequence of nitric oxide-mediated hypovascularity hypoxia development, which affects the early and late downstream stages of systems biology of function, regulation, and homeostasis (Figure 4).

Nitric oxide is necessary for maintaining and sustaining physiological oxygenation during the critical windows of cellular regeneration–rejuvenation and the timing hypothesis of hormone therapy. Physiological concentrations of testosterone significantly increased nitric oxide production (Campelo et al., 2012), and baseline testosterone levels predict body composition and metabolic response to TRT (Deepika et al., 2022) and support the prostate safety of TRT in newly diagnosed men with hypogonadism (Debruyne et al., 2017).

A data-driven generative model suggests a mechanistic explanation for why the selective fitness advantage (bet-hedging) introduced by specific driver genes is tissue-dependent in a tumorigenesis timeline (Lahouel et al., 2020). Hypovascularity hypoxia constitutes the mechanistic interface of a self-perpetuating hypovascularity cyclic/chronic hypoxia dual dynamism CIH–CSH modes (Böger and Hannemann, 2020; Prieto-Lloret et al., 2021) within the pathological tumorigenesis microenvironment niche (Schiffer et al., 2018) (Figure 3).

Endogenous nitric oxide bioavailability and expression is the key gasotransmitter of the NO-cGMP pathway (Liu et al., 2017; Krishnan et al., 2018; Friebe et al., 2020; Feil et al., 2022; Kang et al., 2022). Nitric oxide is involved in the regulation of vasodilation, platelet aggregation, inflammation, hypoxic adaptation, and oxidative stress (Gajecki et al., 2022). Therefore, this insidious early age-related menopause/andropause endogenous nitric oxide-mediated hypovascularity hypoxia development needs further investigation. Emerging genomic evidence from population and experimental studies points to an important role for non-mutagenic promoters in driving cancer incidence rates, and new approaches and research strategies are needed to break this impasse (Brennan and Davey-Smith, 2022). This can provide the needed answers to this important question regarding healthspan for healthy lifestyle aging, cost savings in medical care, and sustainability of the health system.

## Data Availability

The original contributions presented in the study are included in the article/Supplementary Material; further inquiries can be directed to the corresponding author.
